# Response to Lawrence DJ: the global summit on the efficacy and effectiveness of spinal manipulative therapy for the prevention and treatment of non-musculoskeletal disorders: a systematic review of the literature

**DOI:** 10.1186/s12998-021-00380-7

**Published:** 2021-07-20

**Authors:** Pierre Côté, Jan Hartvigsen, Iben Axén, Charlotte Leboeuf-Yde, Melissa Corso, Heather Shearer, Jessica Wong, Andrée-Anne Marchand, J. David Cassidy, Simon French, Gregory N. Kawchuk, Silvano Mior, Erik Poulsen, John Srbely, Carlo Ammendolia, Marc-André Blanchette, Jason W. Busse, André Bussières, Carolina Cancelliere, Henrik Wulff Christensen, Diana De Carvalho, Katie De Luca, Alister Du Rose, Andreas Eklund, Roger Engel, Guillaume Goncalves, Jeffrey Hebert, Cesar A. Hincapié, Maria Hondras, Amanda Kimpton, Henrik Hein Lauridsen, Stanley Innes, Anne-Laure Meyer, David Newell, Søren O’Neill, Isabelle Pagé, Steven Passmore, Stephen M. Perle, Jeffrey Quon, Mana Rezai, Maja Stupar, Michael Swain, Andrew Vitiello, Kenneth Weber, Kenneth J. Young, Hainan Yu

**Affiliations:** 1 Faculty of Health Sciences, Ontario Tech University, Oshawa, Canada; 2 Centre for Disability Prevention and Rehabilitation at Ontario Tech University and CMCC, Oshawa, Canada; 3 Division of Epidemiology, Dalla Lana School of Public Health, University of Toronto, Toronto, Canada; 4 IHPME, Dalla Lana School of Public Health, University of Toronto, Toronto, Canada; 5 Department of Sports Science and Clinical Biomechanics, University of Southern Denmark, Odense, Denmark; 6 Nordic Institute of Chiropractic and Clinical Biomechanics, Odense, Denmark; 7 Intervention & Implementation Research for Worker Health, Institute of Environmental Medicine, Karolinska Institutet, Stockholm, Sweden; 8 ELIB - et liv i bevegelse, Oslo, Norway; 9 Department for Regional Health Research, University of Southern Denmark, Odense, Denmark; 10 Department de Chiropractique, Université du Québec à Trois-Rivières, Trois-Rivières, Canada; 11 Department of Chiropractic, Faculty of Science and Engineering, Macquarie University, Sydney, Australia; 12 Department of Physical Therapy, Faculty of Rehabilitation Medicine, University of Alberta, Edmonton, Edmonton, Canada; 13 Canadian Memorial Chiropractic College, Toronto, Canada; 14 Department of Human Health & Nutritional Sciences, University of Guelph, Guelph, Canada; 15 Rebecca MacDonald Centre, Mount Sinai Hospital, Toronto, Canada; 16 Department of Health Research Methods, Evidence & Impact, Faculty of Health Sciences, McMaster University, Hamilton, Canada; 17 School of Physical & Occupational Therapy, McGill University, Montreal, Canada; 18 Faculty of Medicine, Memorial University of Newfoundland, St. John, ’s Canada; 19 Faculty of Life Sciences and Education University of South Wales, Cardiff, UK; 20 Institut Franco-Européen de Chiropraxie, Ivry-Sur-Seine, France; 21 Faculty of Kinesiology, University of New Brunswick, Fredericton, Canada; 22 Department of Chiropractic Medicine, Faculty of Medicine, University of Zurich & Balgrist University Hospital, Zurich, Switzerland; 23 Department of Anesthesiology, University of Kansas Medical Center, Kansas City, USA; 24 RMIT University, Melbourne, Australia; 25 College of Science, Health, Engineering and Education, Murdoch University, Murdoch, Australia; 26 AECC University College, Bournemouth, UK; 27 Spine Center of Southern Denmark, University Hospital of Southern Denmark, Middelfart, Denmark; 28 Faculty of Kinesiology & Recreation Management University of Manitoba, Winnipeg, Canada; 29 School of Chiropractic, University of Bridgeport, Bridgeport, USA; 30 School of Population and Public Health, Faculty of Medicine, University of British Columbia, Vancouver, Canada; 31 School of Health, Medical and Applied Sciences, CQ University, Sydney, Australia; 32 Stanford University School of Medicine, Stanford University, Stanford, USA; 33 School of Sport and Health Sciences, University of Central Lancashire, Preston, England

Thank you for the opportunity to respond to the Letter to the Editor by Dana J. Lawrence. In his letter, Lawrence states that the results of our systematic review may be due to bias. However, he does not adequately substantiate his claims.

First, Lawrence perceived that we “brushed away” the possibility of reviewer bias. This comment is surprising because, as described in the paper, we carefully planned for possible reviewer bias and took several methodological steps to minimize its potential impact. Specifically, we: 1) developed an instruction manual for critical appraisal that we distributed to all reviewers prior to the Global Summit; 2) used the Scottish Intercollegiate Guidelines Network (SIGN) criteria, a standardized critical appraisal tool, to assess the risk of bias of randomized controlled trials; 3) adapted the language of the SIGN tool to minimize the risk of misinterpretation of the critical appraisal items; 4) provided detailed notes that accompanied the SIGN checklist, and edited to match the purpose of this review; 5) conducted three rounds of independent reviews to ensure that the risk of bias assessment was consistent across reviewers; 6) undertook a quality control assessment by two independent reviewers, who reviewed all critical appraisal checklists and risk of bias ratings; and 7) invited all Global Summit participants to independently review and vote on the final risk of bias assessment results; the risk of bias table was approved by 98.0% (49/50) of participants. Therefore, the position advanced by Lawrence that we “brushed away” the possibility of reviewer bias is not justified.

Second, Lawrence states that publication bias may be responsible for our findings. He wrote: “*… publication bias tilted toward positive, not negative, findings have been demonstrated even in Cochrane reviews themselves.”* and he uses the study by Kicinski et al. to support his assertion [1]. This indicates that Lawrence misinterpreted the concept of publication bias in this instance. In their study of systematic reviews included in the Cochrane database, Kicinski et al. reported that: “In the meta-analyses of efficacy, outcomes favoring treatment had on average a 27% (95% Credible Interval (CI): 18% to 36%) higher probability to be included than other outcomes.” [1] We refer Lawrence to our discussion where we clearly stated: “Finally, publication bias may be present in this field of research. However, it is unlikely that publication bias compromised the validity of our results because studies most unlikely to be published are those that failed to obtain a ‘positive’ result.” In other words, we are in agreement with Kicinski et al. and disagree with Lawrence’s conclusion. In fact, our position is consistent with a large body of methodological literature which clearly indicates that a trial is more likely to be published if the results favor a specific intervention (positive results) compared to a trial which does not support an effect of an intervention (negative results) [1–13]. We would like to remind Lawrence that we identified and critically appraised studies regardless if they had “negative” or “positive” results. However, the methodological quality of “positive” trials was low and their results could not be used to inform our synthesis.

Third, Lawrence indicated that clinicians did not have a voice in the project. We respectfully disagree with this assertion. Several participants to the Global Summit maintain an active practice and most participants have had long and successful clinical careers.

In summary, the statements made by Lawrence about our methodology are incorrect and ill informed. While we thank Lawrence for his interest in our work, his statements about our methodology are in our opinion misconstrued and hence not appropriate.

## Data Availability

Not applicable.
